# Infant Cry Signal Diagnostic System Using Deep Learning and Fused Features

**DOI:** 10.3390/diagnostics13122107

**Published:** 2023-06-19

**Authors:** Yara Zayed, Ahmad Hasasneh, Chakib Tadj

**Affiliations:** 1 Department of Natural, Engineering and Technology Sciences, Faculty of Graduate Studies, Arab American University, Ramallah P.O. Box 240, Palestine; y.zayed1@student.aaup.edu; 2 Department of Electrical Engineering, École de Technologie Supérieur, Université du Québec, Montréal, QC H3C 1K3, Canada; chakib.tadj@etsmtl.ca

**Keywords:** infant’s crying diagnosis, audio domains features, HR, GFCC, spectrogram, deep learning, machine learning

## Abstract

Early diagnosis of medical conditions in infants is crucial for ensuring timely and effective treatment. However, infants are unable to verbalize their symptoms, making it difficult for healthcare professionals to accurately diagnose their conditions. Crying is often the only way for infants to communicate their needs and discomfort. In this paper, we propose a medical diagnostic system for interpreting infants’ cry audio signals (CAS) using a combination of different audio domain features and deep learning (DL) algorithms. The proposed system utilizes a dataset of labeled audio signals from infants with specific pathologies. The dataset includes two infant pathologies with high mortality rates, neonatal respiratory distress syndrome (RDS), sepsis, and crying. The system employed the harmonic ratio (HR) as a prosodic feature, the Gammatone frequency cepstral coefficients (GFCCs) as a cepstral feature, and image-based features through the spectrogram which are extracted using a convolution neural network (CNN) pretrained model and fused with the other features to benefit multiple domains in improving the classification rate and the accuracy of the model. The different combination of the fused features is then fed into multiple machine learning algorithms including random forest (RF), support vector machine (SVM), and deep neural network (DNN) models. The evaluation of the system using the accuracy, precision, recall, F1-score, confusion matrix, and receiver operating characteristic (ROC) curve, showed promising results for the early diagnosis of medical conditions in infants based on the crying signals only, where the system achieved the highest accuracy of 97.50% using the combination of the spectrogram, HR, and GFCC through the deep learning process. The finding demonstrated the importance of fusing different audio features, especially the spectrogram, through the learning process rather than a simple concatenation and the use of deep learning algorithms in extracting sparsely represented features that can be used later on in the classification problem, which improves the separation between different infants’ pathologies. The results outperformed the published benchmark paper by improving the classification problem to be multiclassification (RDS, sepsis, and healthy), investigating a new type of feature, which is the spectrogram, using a new feature fusion technique, which is fusion, through the learning process using the deep learning model.

## 1. Introduction

Even though the worldwide number of infant deaths has decreased from 5 million in 1990 to 2.4 million in 2019, newborns still suffer the highest risk of mortality during the first 28 days of life. In 2019, neonatal deaths accounted for 47 percent of all deaths among children under the age of 5, with nearly one-third dying on the day of birth and nearly three-quarters dying during the first week of life [1]. Infants who die within the first 28 days of life are afflicted with illnesses and problems due to a deficiency in the quality of care during delivery or professional care and treatment shortly after birth and in the early days of life [2]. This demonstrates that newborns are vulnerable to a variety of diseases that might result in lifelong illnesses or early death. Some of these diseases are aspiration, asphyxia, kidney failure, RDS, and sepsis. RDS and sepsis are the most common pathologies associated with a high mortality rate; thus, this research study started by diagnosing them at early stages.

RDS is considered the major cause of death and illness among preterm newborns [3]. RDS is a respiratory disorder of neonates that manifests itself immediately after delivery. It is one of the most frequent reasons for newborn intensive care unit admissions (NICU) and breathing failure in newborns [4]. Some of the causes of this disease are maladaptation or delayed adaptation, a preexisting condition such as surgical or congenital defects, and acquired infections, which are all causes of developmental delay [4]. RDS caused deaths at a rate of 10.7 per 100,000 live births in the United States in 2020 [5]. The diagnosis of RDS requires a set of clinical tests including chest X-ray, computerized tomography (CT), electrocardiogram, and echocardiogram for the heart and frequent blood tests to monitor the oxygen levels [6].

Moreover, sepsis is a significant source of death and disease. It caused 15 deaths per 100,000 live births in the United States in 2020 [5]. The main criterion in the diagnosis of sepsis is the isolation of the pathogen in one or more blood cultures [6]. However, it is not easy to grow the pathogenic microorganism in culture in all cases because of many reasons, including inadequate sample collection, slow-growing microorganisms, prior antimicrobial therapy, nonbacterial infections, and contamination. In addition to that, and like RDS, sepsis needs a set of tests to be diagnosed which is related to heart rate, feeding problems, lethargy, fever, hypotonia, convulsion, hemodynamic abnormalities, and apnea [7]. Early detection of these hidden illnesses, such as sepsis and RDS, is critical. As most of the newborns who are infected by such pathologies seem normal at birth, and as can be seen from both RDS and sepsis, these pathologies need a lot of clinical tests that are time-consuming in addition to the risk of them resulting in false-negative and false-positive outputs [8]. Thus, early detection of hidden illnesses for prompt and successful treatment within the first week of life is critical, as it might save these newborns’ lives [9].

On the other hand, the only way infants can communicate with their surroundings is by crying. Through training and experience, experts such as experienced parents, pediatricians, and childcare professionals might be able to understand and distinguish the meaning of infants’ crying. However, interpreting newborn screams may be challenging for new parents as well as unskilled clinicians and caregivers. As a result, distinguishing infants’ cries with distinct meanings based on related cry audio qualities is critical [10]. Accurately interpreting newborn cry sounds and automatically identifying infant cry signals may assist parents and caregivers in providing better care to their infants. Early diagnosis of diseases via cry signals is noninvasive and may be conducted without the presence of specialists; hence, it has the potential to save more lives, particularly in undeveloped countries [11].

Researchers discovered in the early years that distinct sorts of cries may be distinguished aurally by trained adult listeners [12]. However, teaching human perception of newborn screams is much more difficult than training machine learning (ML) models, which shows a promising result in newborn cry signal detection and classification [13]. In recent years, this field of infant crying analysis has received great attention, where researchers aim to employ ML techniques for newborn crying analysis and diagnosis, in addition to speech processing approaches, whether based on time or frequency domains, to develop a diagnostic system that can diagnose infant diseases in their early stages. Infants’ crying could be related to the infection of one or more well-known infant diseases, such as sepsis, fever, deaf, autism, vomiting, meningitis, kidney failure, respiratory distress syndrome (RDS), asphyxia, jaundice, premature, etc., [9,14]. The cries of pathological newborns with neurological disabilities have special characteristics such as high-pitched cries and prolonged cries, and they are more irritable and difficult to console [15]. Consequently, CAS analysis and classification may be used as a beneficial technique for predicting and identifying newborn illnesses before the onset of symptoms. Various feature categories, such as cepstral, prosodic, spectrogram, etc., may be calculated and created utilizing audio signals of crying.

In this research work, we aim to propose an automated diagnostic machine learning model that depends on crying signals to diagnose different newborns’ diseases, specifically the sepsis and RDS pathologies, as well as to distinguish the normal healthy crying signals. The proposed model is based on extracting and modeling more efficient features from different voice domains, such as image-based, prosodic, and cepstral features, which is different from the very recently published work [8], which used only the last two domains. The ultimate goal of this system is to provide assistance to the medical clinics and parents to understand and diagnose the crying of the infants, thus taking the correct actions if the system indicates that the crying is related to a certain pathology. Moreover, it will prevent misunderstanding between the parents and caregiver, which subsequently reduces their stress. Furthermore, the system will be able to diagnose the baby without overwhelming him/her with extensive medical tests [16]. This will result in tackling many medical problems where a diagnosis is provided based on invasive procedures, allowing for early detection [16].

The rest of this paper is organized as follows: Section 2 presents a literature review of the research topic. Section 3 illustrates the proposed model and the methodology. Afterward, the experiments and results are presented in Section 4 and discussed in Section 5, followed by the conclusion and future work for this research field in Section 6.

## 2. Literature Review

Numerous research works have been conducted to detect infant crying [17,18,19] and to identify the reason behind this crying and if this is related to a pathological case. Most of the current research works have focused on classifying pathological from healthy infants, using crying cues [20]. Other works go into more specifics to diagnose certain pathologies such as hypoacoustic [21], asphyxia [22,23,24], hypothyroidism [25], septic [18], RDS [26], and autism spectrum disorder (ASD) [27]. Such research studies and systems mainly involved two main stages, the feature computation and extraction stage, using the CAS and based on different audio domains, including the cepstral domain features, prosodic domain features, image domain features, time domain features, and wavelet domain [14]. The computed features are fed into the next part of the ML model which could be traditional machine learning models or DL models since researchers have recently begun to explore the use of DL algorithms for analyzing infant crying. DL approaches have shown effective results in automatically extracting useful features from audio signals and in classifying sounds into different categories such as healthy and sick infants [19,22,24,28,29,30,31,32].

Most researchers have adopted the cepstral domain features in the feature extraction from audio signals such as Mel frequency cepstral coefficients (MFCC) [33,34,35,36], linear frequency cepstral coefficients (LFCC) [37], short-time cepstral coefficients (STCC) [37], and Bark frequency cepstral coefficients (BFCC) [38], combined with both DL and traditional ML models. MFCCs were the most used in identifying infant pathologies. For instance, in [33], the authors’ system was used to classify the causes of the infants’ crying into eight reasons, including belly pain, discomfort, hungry, sleepy, and tired. The MFCC coefficients have been used to train three ML algorithms, including the K-nearest neighbors rule (KNN), SVM, and naïve Bayes classifier (NBC). The KNN had the highest accuracy of 76%. In [34], they used a dataset of CAS for healthy and pathological infants including 34 pathologies. As a first step, feature extraction was performed using a different set of techniques including the extraction of MFCC and amplitude modulation features. These features were fed into two machine learning algorithms, probabilistic neural networks, and an SVM algorithm with an accuracy of 72.80% and 78.70%, respectively.

Moreover, the MFCC was adopted for feature extraction from audio signals [28] to be used in the training of set machine learning models, including artificial neural network (ANN), CNN, and long short-term memory (LSTM). These ML models were trained to achieve two purposes, identify sick and healthy babies, and then determine the baby’s needs such as hunger/thirst, need for a diaper change, and emotional needs. On the first goal, CNN was able to achieve an accuracy of 95% and an accuracy of 60% was achieved for the second classification purpose. A similar feature extraction was also used along with KNN in [35] and achieved an accuracy of 71.42% in determining the reason for crying, including hunger, belly pain, need for burping, discomfort, and tiredness. In [36], MFCC was used with the CNN model with multiple variants to test and multistage a heterogeneous stacking ensemble model, which consists of four levels of algorithms, Nu-support vector classification, random forest (RF), XGBoost, and AdaBoost. The classification results of the CNN model outperformed the other ML algorithms, reaching an accuracy of 93.7%.

The prosodic domain features were also employed in the analysis and diagnosis of infants’ crying signals. This domain includes much valuable information, such as variations in intensity, fundamental frequency (F0), formants, harmonicity, and duration, which contribute a lot to infant crying signals analysis. This has been followed by a lot of research regarding whether stand-alone or being combined with the cepstral features improves performance. For instance, in [39], they based the proposed model on mean, median, standard deviation, and minimum and maximum of F0 and F123 to distinguish between full-term and preterm infant cries. In contrast, in [22], they used a combined model of weighted prosodic features and MFCC features, thus feeding them into a DL model which was able to achieve a 96.74% accuracy. The obtained results emphasized the importance of using both domains in extracting and modeling a more efficient feature set.

The authors in [40] depended on the wavelet domain audio feature by using the discrete wavelet transform (DWT) method to extract the coefficient characteristics. These coefficients have been used in the classification process using a single-layer neural feed-forward (SLNF) network. This system was able to distinguish between five categories of crying: Eh, Eairh, Neh, Heh, and Owh. Each one is related to a specific condition in a baby, where Heh is related to the feeling of discomfort, and Owh is related to feeling sleepy. Neh indicates thirst or hunger, and Eairh is related to the feeling of burping due to congested air in the chest or stomach. The crying signals were passed through discrete wavelet transform for feature extraction where all signals were then extracted for cry classification using five scaling functions of the wavelet transform, namely Haar, Db2, Coif1, Sym2, and Bior3.1, where the output of each function is used as an input for SLNF. The average accuracy of all discrete wavelet functions on the baby language is over 80%.

Furthermore, the image domain features were used in this field of study, where the main feature is the spectrogram, which is an image or a time–frequency representation of audio [14]. For example, the researchers in [32] classified the neonatal cry signals into pain, hunger, and sleepiness, using the short-time Fourier transform (STFT) technique to generate the spectrogram images, which were used as an input for training a deep convolutional neural network (DCNN), where the extracted features from the DCNN were used as an input for the SVM classifier, which was able to reach an accuracy of 88.89% using the radial basis function (RBF) kernel. Similarly, the spectrogram for the feature extraction and SVM classifier obtained an accuracy of 71.68% [41]. Moreover, the researcher in [29] used the spectrogram with the CNN model for classifying the condition of the baby, whether sleepy or in pain, and obtained an accuracy of 78.5%.

Some researchers have gone more deeply into this topic to diagnose a specific disease. For instance, the authors in [42] suggested a machine learning model to diagnose hypoxic ischemic encephalopathy disease in newborns based on CAS analysis. Multiple feature extraction techniques were used, including the MFCC and Gammatone frequency cepstral coefficients (GFCCs). These features were utilized by a basic deep network, achieving an accuracy of 96%. The authors in [37] introduced a classification model between healthy and unhealthy newborn cries. A set of feature extraction techniques were used, including MFCC, LFCC, STCC, and Teager energy cepstral coefficients (TECC). The classification process is based on the Gaussian mixture model (GMM) and SVM algorithms. Both models have been trained using the different features extracted separately and the results justified the superiority of the TECC representations with the GMM classifier, which achieved an accuracy of 99.47%. Furthermore, in [31], the researchers developed a DL approach that can classify healthy and pathological babies based on the infant’s CAS, where the signals were processed using cepstrum analysis to extract the harmonics in the cry records, and the outputted spectrum was fed into three DL models including deep feed-forward neural networks (DFFNN), LSTM, and CNN. The latter DL model outperformed the other algorithms with an accuracy of 95.31%. Similarly, the researchers in [43] adopted the cepstrum to build a model to distinguish between healthy and pathological infants based on the crying signal by evaluating DFFNN, naïve Bayes, SVM, and a probabilistic neural network. The DFFNN achieved a 100% accuracy.

Few researchers have followed a combined features domain similar to the work in [8] where they combined both GFCC and HR features by using simple concatenation to distinguish between RDS and sepsis. Using SVM and MLP, the SVM achieved 95.29% compared to 92.94% for the GFCC alone and 71.03% for the HR. While in [44], they combined images that contain the prosodic feature lines including F0, intensity, and formant spectrogram CNN and waveform CNN, producing a 5% better accuracy. This study [45] explored the use of DL models with hybrid features to classify asphyxia cries in infants. The models used a combination of MFCC, chromagram, Mel-scaled spectrogram, spectral contrast, and Tonnetz features. The results showed that the DNN models performed better with the hybrid features, achieving a 100% accuracy for normal and asphyxia cries, and a 99.96% accuracy for nonasphyxia and asphyxia cries. The CNN model performed better with the MFCC alone. The study demonstrated the effectiveness of using DL models with hybrid features for classifying asphyxia cries in infants.

Despite the existence of these related research works, the question of constructing the optimal feature set for the problem of classifying different pathological infant crying signals remains open and needs further investigation. In addition, most of the existing works have mainly focused on identifying one pathology [21,22,23,24,25,26,27] by using different machine learning techniques or identifying two pathologies at most [8]. Moreover, there is only little attention paid to combining different feature domains of the CAS using a simple concatenation technique [8]. The main contributions of this paper are threefold: (1) The use of combined DL models to extract more efficient features that could sparsely discriminate between classes of the infant’s pathological signals in the feature space, and thus simplify and improve the linear separation between these pathologies. (2) The investigation of feature fusion and modeling of three different audio domains, including the cepstral domain, the prosodic domain, and the spectrogram image domain. The feature fusion process itself was investigated using the classical feature concatenating process before feeding them into the training network, and by relying on fusing the different features within the learning process. (3) Distinguishing between two pathological cases (sepsis and RDS) and the healthy case using different ML and DL approaches that were fine-tuned to produce the best classification rates, where the fine-tuned and combined DL model obtained an accuracy of 97.50% to distinguish between sepsis, RDS, and the healthy crying cases.

## 3. Methodology

**Figure 1:**
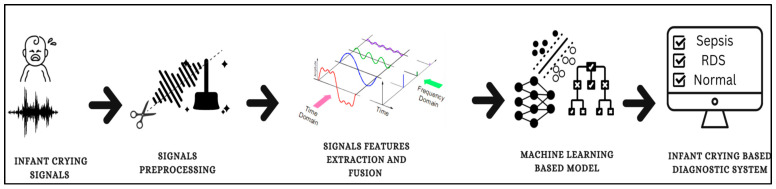
The workflow of a machine learning-based model for classifying infant pathological cues.

Mainly, developing a diagnostic system based on CAS includes several main steps, as shown in Figure 1, including CAS acquisition, signal preprocessing and preparation, feature extraction using one or more audio domains, followed by feeding the extracted feature into a machine learning classifier, where the outcome is a diagnostic system that can distinguish between infant pathological cues.

### 3.1. Data Acquisition

The data used in this research work were acquired from both Saint-Justine Children’s Hospital in Montreal, Canada, and the Al-Raee and Al-Sahel hospitals in Lebanon. They have been used previously in many similar kinds of research work [8,31]. The dataset contains samples of crying audio for newborns, aged 1–53 days, with different demographic characteristics, as shown in Table 1. These signals were recorded using a common digital 2-channel Olympus handheld recorder with a 16-bit resolution and 44,100 Hz sampling frequency placed in the 10-to-30 cm vicinity of the newborns. The pathological status of the infant was identified based on medical tests and reports and the signals were labeled as pathological with the specific pathology or normal crying. Note that the dataset includes many types of pathologies, such as RDS, kidney failure, aspiration, asphyxia, and sepsis, in addition to the healthy case. The original recordings had an average duration of 90 s. This was performed 5 times for each newborn. To overcome the limited number of recorded samples, which is attributable to several factors such as the unpredictability of whether a newborn with the targeted pathology groups will be observed during the data collection period, acquiring the ethical and technical approvals to incorporate a cry sample in the database is a timely and difficult process which may result in losing some of the samples and obtaining the newborns’ guardians’ consent to record their newborn’s cry and then adding it to the database is quite challenging. Given all these obstacles, we tried to segment each recording into multiple expiration segments in order to overcome the data limitation challenge and to better study the characteristics of pathological newborn cries. The segmentation process was applied in the next step to generate multiple expiration (EXP) segments, then these data were randomly sampled to select an equal number of samples from each category of 1132, similar to the research study in [8], which is used as a benchmark for our research to guarantee a fair comparison. The created segmented dataset is a balanced and homogeneous dataset of a total of 3396 records, as shown in Table 2.

### 3.2. CAS Preprocessing

The preprocessing steps were applied by previous researchers [8,9,26,34,43,46]. The data were preprocessed to eliminate background noise, artifacts, and silence, and segment each audio. After segmentation, each audio recording was labeled with multiple labels (EXP, INSV). The EXP label represents the expiratory cries, while the INSV stands for phonation during inspiration, which represents a voiced inspiratory cry segment. WaveSurfer software was used to perform the segmentation process. In this study, the expiratory data were used, where each resulting segment is considered as a sample. This segmentation process was able to solve the issue of the limitation of the data, which could affect the ability to identify the pathology, by reducing the length of the recorded audio signals to 90 s, which facilitates and speeds up the training process. The samples which were less than 17 s were excluded as they were noninformative recordings that may have disturbed the training process.

### 3.3. CAS Feature Extraction and Fusion

Feature extraction is a critical stage in implementing the classification model as it impacts the classification rate and reliability of pattern recognition. In this study, the feature extraction process was performed by considering the short-term representations through the GFCC features and the spectral representation of the signals through the harmonic ratio. These features were extracted using MATLAB code. In addition, the image domain features were utilized by generating the spectrogram images using Python libraries and convolutional neural networks to extract the features from those images. The samples which were less than 17 s were excluded as they were noninformative recordings, as stated above. As an additional step, the data were normalized using the standard scaler before being fed into the training phase using the following equation.

(1) $$z=\frac{x\;-\;\mu }{\sigma },\;$$

where x is the value, μ is the mean and σ is the standard deviation.

### 3.4. CAS Classification Model

In this study, three ML classification models have been experimented with, specifically SVM, RF, and DNN. These models were chosen due to their ability to handle complex features, which is essential for accurately diagnosing these conditions. SVM has been used for infant cry classification in a lot of research [47] because it can handle nonlinear relationships between the acoustic features and the different cry categories. The RF was used for such systems [48] because it is robust to noise in the data and can handle high-dimensional feature spaces. DNNs are a relatively new and powerful class of models that can effectively capture complex patterns and fuse the features during the learning process, enabling better classification accuracy. One of the main contributions of this paper is fusing through the learning process, so the DNN was the best choice to do so. We aim to evaluate the performance of these models and compare them to other available models in the literature.

#### 3.4.1. Support Vector Machine (SVM)

SVM is one of the supervised learning techniques for classification [49], regression [50], and outlier detection [51]. SVM is a classifier that works by creating a hyperplane or multiple hyperplanes for separation, which implies giving the training data labels based on the optimal hyperplane that will categorize the new sample [52]. SVMs are widely used as probabilistic classifiers for classifying newborn cries, using various SVM models such as multiclass SVMs, linear and RBF kernel-based binary SVMs, and incremental SVM learning models. SVMs are highly dependent on the selection of hyperparameters such as C, gamma, and kernel type. The regularization parameter C controls the trade-off between the model complexity and training error, while the kernel type determines the nonlinear mapping between the input and feature space. The kernel coefficient gamma controls the influence of each training example in the decision boundary. These models are designed to continuously add new data to the training set at each training step, and they typically utilize both prosodic and temporal features as the input [11]. The SVM classifier is one of the prevalent algorithms when it comes to newborn cry applications; hence, it is often used as a baseline in several studies to illustrate the significance of subsequent phases of the design. This is because data in biological investigations are sometimes quite restricted, and one of SVM’s greatest strengths is its ability to create complicated decision boundaries effectively from small samples.

#### 3.4.2. Random Forest (RF)

Random forest is also a supervised classifier that can be used for both regression and classification. RF works by creating a set of decision trees using the training data and then predicting the output for the unseen data based on the accuracy and majority vote. The RF algorithm has a set of hyperparameter criterion, which is the split quality measurement function; max_depth, which is the maximum depth for the tree; min_samples_leaf, which is the minimum sample number to decide a leaf; min_samples_split, which is the minimum sample number to decide a split; and n_estimators, which is the number of decision trees to be built on the RF [53]. RF was used in newborn crying signal analysis in much research [48].

#### 3.4.3. Deep Learning Approach

**Figure 2:**
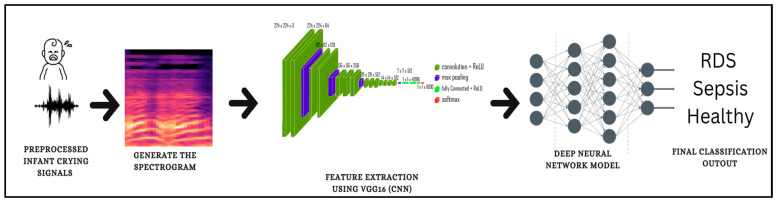
Spectrogram and Deep Learning Classification Methodology.

Deep learning neural networks are a type of machine learning algorithm that is modeled on the structure and function of the human brain. They consist of multiple layers of interconnected nodes, or “neurons,” as shown in Figure 2. Those nodes receive inputs, apply mathematical operations, and produce outputs. These outputs are then passed on to other neurons in subsequent layers of the network, where the process is repeated until a final output is produced. DL neural networks have been shown to be particularly effective in tasks such as image and speech recognition [54], natural language processing [55,56], and time series prediction [57]. Deep learning models are a type of artificial neural network that is capable of learning complex patterns in data. These models have many hyperparameters that need to be carefully tuned for optimal performance. Some of the most important deep learning parameters include the number of layers, the number of neurons in each layer, the activation function, the learning rate, and the number of epochs. The number of layers and neurons affects the complexity of the model, while the activation function determines how the output of each neuron is calculated. The learning rate controls the step size taken during the gradient descent, and the number of epochs determines the number of times the model will be trained on the entire dataset. Finding the right combination of hyperparameters for deep learning models can be a challenging task but is essential for achieving high accuracy on a given task.

The spectrogram images are used as the input for the VGG16 pretrained model to extract the most important features to be used later on in the classification process, as shown in Figure 2. As these images are large, this pretrained convolutional neural network model was used to extract the features. VGG 16 is a simple CNN that serves as a foundational example of a CNN design [58].

The VGG16 model was pretrained on the ImageNet dataset, which is a large-scale dataset consisting of millions of labeled images from various object categories. The ImageNet dataset has been widely used as a benchmark for image classification tasks. It encompasses a wide range of object classes, including animals, plants, vehicles, and everyday objects.

**Figure 3:**
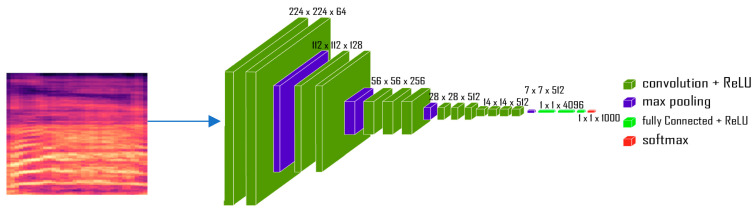
VGG16 pretrained CNN model [60].

The VGG16 architecture, as shown in Figure 3, is a CNN with 16 layers. The input to the network is an image with 3 channels (RGB) and a size of 224 × 224 pixels. The first layers of the network consist of 2 convolutional layers with 64 filters each, followed by a max pooling layer. This is repeated two more times, resulting in four blocks of convolutional and pooling layers. The next 4 blocks consist of 3 convolutional layers with 128, 256, and 512 filters, respectively, followed by a max pooling layer. The final block consists of 3 convolutional layers with 512 filters each. After the convolutional layers, the network has a fully connected (dense) layer with 4096 units, followed by a dropout layer to prevent overfitting. This is followed by another dense layer with 4096 units. This layer is commonly used as a feature extractor in transfer learning, as it provides a high-level representation of the input image that can be used for classification. Additionally, the 1000 layer in the VGG16 outputs a vector of 1000 elements, representing the confidence of the model in predicting the presence of objects or concepts in an image. Therefore, these 4096 features will be used as an input layer on the DL network [59].

DL models were used in a few infant crying analyses [19,22,28,29,31,32]. They are able to automatically learn and extract features from the input data and can handle large and complex datasets, as it applies dimensionality reduction during the learning process. In addition to that, DL can extract the most efficient and sparse features, which makes it very efficient in classification problems. However, training DL neural networks can be computationally expensive, and it requires a large amount of labeled data. In this study, we chose to use Apple M1 chip with a Pro GPU for training our DL model due to its faster computation time and higher processing power compared to traditional CPUs. The use of GPU enabled us to train our DL model in a fraction of the time it would have taken using a CPU, run more experiments, and fine-tune our model more efficiently. We also observed that the model achieved better performance on the GPU compared to the CPU, with a noticeable increase in accuracy and a decrease in training time.

In addition to the ML and DL algorithms, the GridSearchCV technique is used to optimize the hyperparameters, where these parameters are used to configure the model [61] by picking up the value of the parameter that achieves the highest performance by experimenting with a set of given values. This method guarantees a much better performance than the models implemented using randomly selected values for the parameters [62]. The GridSearchCV also applies cross-validation through training. As such, the dataset is divided into k sets. Through each iteration, one part is specified for testing and the other k-1 parts for training, and so on. At each iteration, the performance is recorded, and the result is the average of all these values. The Keras tuner library was used to tune the DNN model which is an open-source library for optimizing machine learning models. It is built on top of the Keras library and allows for performing hyperparameter tuning on models easily and efficiently.

Many performance indicators are used to evaluate the trained models, including the accuracy, receiver operating characteristic (ROC), and confusion matrix, including the false positive (FP), false negative (FN), true positive (TB), true negative (TN), accuracy, precision, recall, and F1-score. Table 3 below shows a number of these measures used in this study.

Accuracy measures the overall correctness of the model’s predictions. In this case, it is the percentage of all correctly classified instances out of the total number of instances, while precision measures the proportion of correct predictions among all positive predictions made by the model. In the context of RDS, sepsis, and normal classification, precision would be the proportion of correctly predicted RDS, normal, or sepsis cases out of all the predicted RDS, normal, or sepsis cases. A high precision score means that the model makes fewer false-positive predictions. Recall measures the proportion of actual positive instances that are correctly predicted as positive by the model. In the context of RDS and sepsis classification, recall would be the proportion of correctly predicted RDS, normal, or sepsis cases out of all the actual RDS, normal, or sepsis cases. A high recall score means that the model makes fewer false-negative predictions. The F1-score combines precision and recall into a single score by calculating their harmonic mean. It provides a balanced measure that considers both false positives (precision) and false negatives (recall). In this problem, a higher F1-score would indicate that the model is performing well in terms of both accuracy and its ability to correctly classify RDS, sepsis, and healthy cases while minimizing false positives and false negatives.

## 4. Experiments and Results

### 4.1. GFCC and HR Features Simple Concatenation

**Figure 4:**
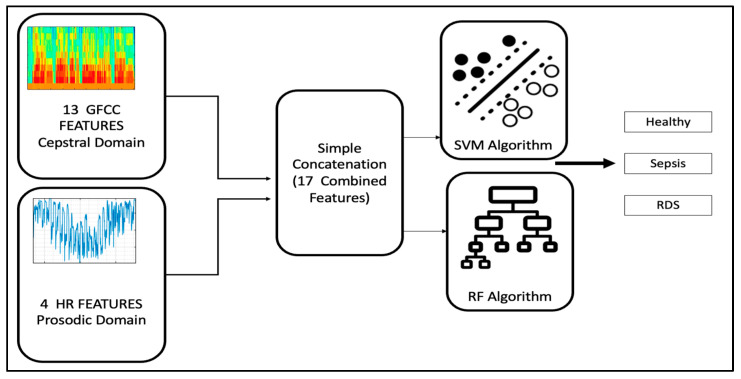
GFCC and HR simple concatenation combination.

The first part of the study used the same methodology followed by the published research [8] where they investigated both the GFCC and HR features, whether separately or combined, using a simple concatenation with an additional class. The published paper investigated only sepsis and RDS, but our study introduces the normal crying category as a step to implement a wider diagnosing system in the future. Each feature has been evaluated separately, then the GFCC and HR features are combined by a conventional concatenation and fed into the SVM and RF, as shown in Figure 4.

Initially, the ML algorithms were applied with their default parameters without any hypertuning. This was performed on the GFCC features, HR features, and the combined GFCC and HR features. Table 4 shows the accuracy result for each model.

**Figure 5:**
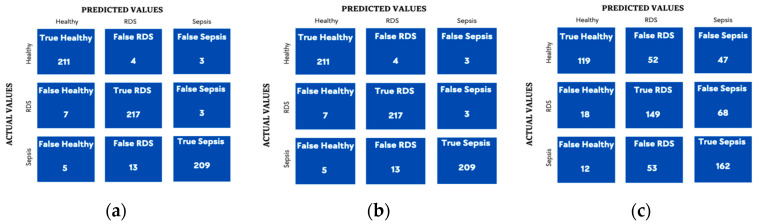
Confusion matrix for Hyper-tuned SVM algorithm. (**a**) Combined Features; (**b**) GFCC Features; (**c**) HR Features.

**Figure 6:**
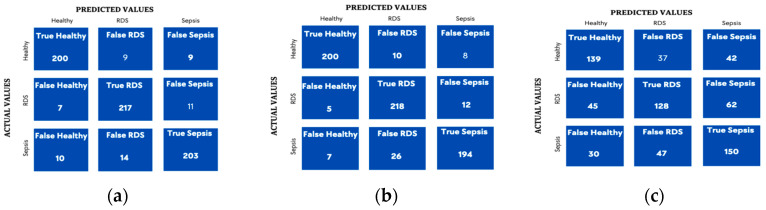
Confusion matrix for hyper--tuned RF algorithm. (**a**) Combined Features; (**b**) GFCC Features; (**c**) HR Features.

The models are hypertuned to guarantee the best performance by choosing the optimal combination of hyperparameters for each model. Table 5 and Table 6 show the optimal combination chosen by the GridSearchCV for each parameter, followed by confusion matrices in Figure 5 and Figure 6 and the table of the precision, recall, F1-score (See Table 7), as well.

**Figure 7:**
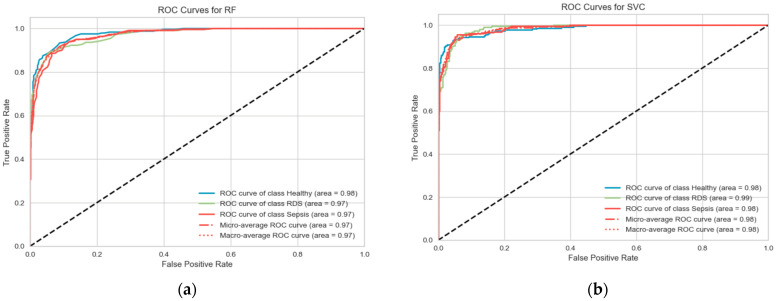
Tuned ROC curve for combined features, including GFCC and HR features. (**a**) Random Forest; (**b**) SVM.

To draw a clearer image of the result, the ROC curve in Figure 7 was plotted but as we were dealing with a multiclassification problem, the multiclass ROCAUC curves were used from a library called Yellowbrick, as the sklearn ROC curve is only used for binary classification problems. Therefore, the library handles this by addressing this by binarizing the output (per class) or using one-vs-rest (micro score) or one-vs-all (macro score) strategies of classification [63]. The plots in Figure 7 below for both the SVM and RF show the ROC curve for the three categories (RDS, sepsis, healthy) in the three datasets combined, GFCC and HR, respectively.

**Figure 8:**
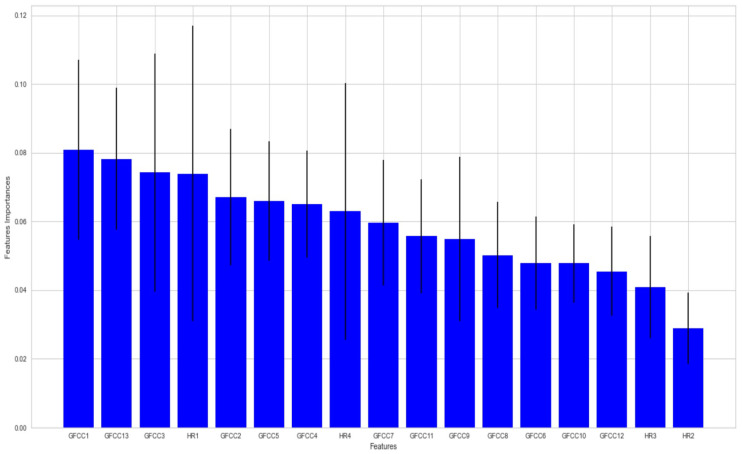
Feature Importance for GFCC and HR Features in Random Forest Model.

To confirm the importance of the features of both GFCC and HR, the feature importance plot was generated in Figure 8.

### 4.2. Spectrogram, HR, and GFCC Fusion (Through Input and through the Learning Process)

**Figure 9:**
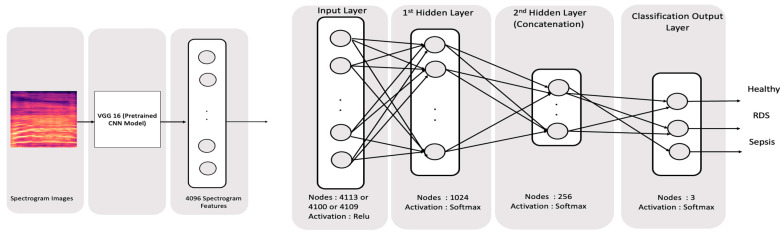
Concatenation through the input for Spectrogram, GFCC, and HR features. The input layer number of nodes is 4113, 4109, or 4100 (corresponding to (1) spectrogram, GFCC, and HR, (2) spectrogram and GFCC, and (3) spectrogram and HR).

In the second part, the DNN is used to investigate a new feature type, which is the spectrogram, as it contains thousands of features that could be beneficial to the classification process and appropriate for DNN models. A complete structure was used in order to make a reduction in features as we have a large number of features. Two feature fusion techniques were experimented with in this section where, initially, the spectrogram was fused with the other features on the input layer before being fed into the DNN network of the structure that contains four layers of DNN. The first has the input layer that has 4096 nodes for the spectrogram added to 13 GFCC nodes, which totals 4109 nodes, or both the GFCC and HR features which create 4113 nodes, followed by 2 fully connected layers in sizes of 1024 and 256 that use the relu activation function. The 256 features are used to calculate the output layer of 3 nodes using the SoftMax activation function. Figure 9 below shows the full topology for the DNN network.

To confirm the ability of the DL model to extract the most important features, we combined the spectrogram, HR, and the GFCC and fed them into the SVM model directly. This is considered a comparison between a sophisticated model and a statistical model (Softmax). The 3 architectures are trained using 120 epochs within approximate 80 s.

The SVM model was hypertuned using the GridSearchCV, while the Keras tuner library, which is an open-source library for optimizing machine learning models, was used to tune the DNN model. It is built on top of the Keras library and allows you to perform hyperparameter tuning on your models easily and efficiently. Table 8 and Table 9 below show the values for the parameters that were tuned. The results of the simple concatenation experiment are shown in Table 10 below.

**Figure 10:**
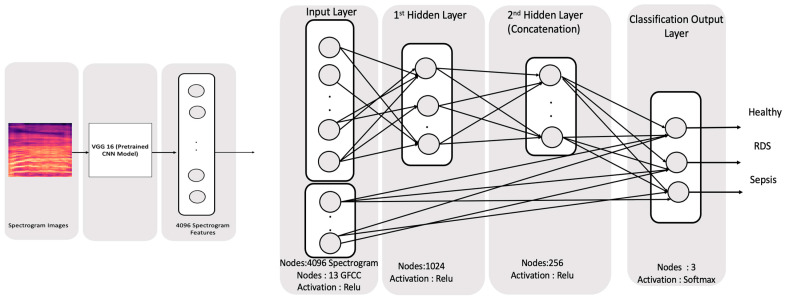
Concatenation through the input for Spectrogram and GFCC.

The result of the simple concatenation shows a little improvement in the accuracy so another fusion technique was used by fusing the features through the learning process which would improve the effect of the features on the classification process. The used DNN network contains a four-layer DNN. The first has the input layer of 4096 features for the spectrogram followed by 2 fully connected layers of size of 1024 and 256 that use the relu activation function. The 256 features fused with the 13 GFCC features are used to calculate the output layer of 3 nodes using the SoftMax activation function. Figure 10 below shows the topology for the fusion through learning the DNN network. It was trained using 100 epochs within 73 s.

**Figure 11:**
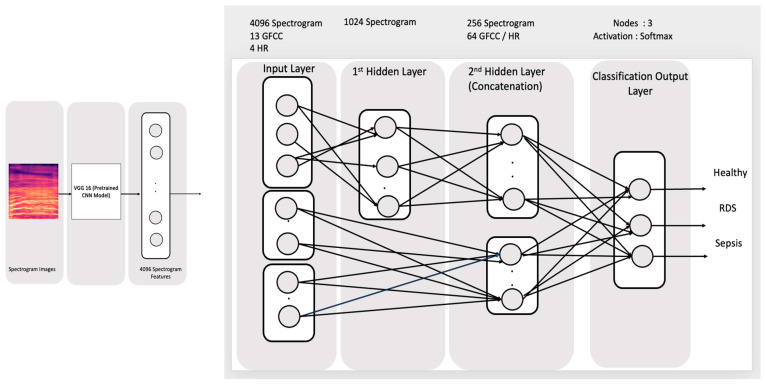
Concatenation through the input for 4096 Spectrogram, HR, and GFCC through learning.

Similar to the previous topology, the GFCC has been fused with the HR features into a 64 nodes layer, which is afterward used to calculate the output layer along with the 256 nodes calculated with the spectrogram. This model is trained using 80 epochs through 90 s. The topology of the model is shown in Figure 11.

**Figure 12:**
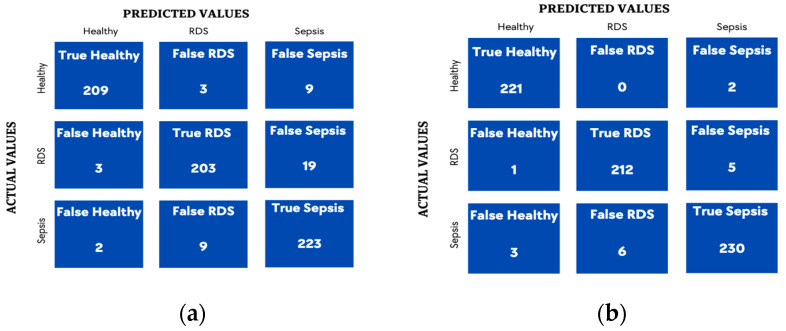
Tuned Confusion Matrices for combined features spectrogram, HR, and GFCC. (**a**) SVM; (**b**) DNN through learning fusion for the model presented in Figure 11.

**Figure 13:**
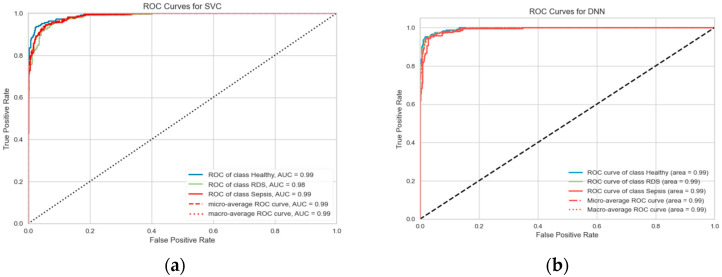
Tuned ROC curve for combined features spectrogram, HR, and GFCC. (**a**) SVM; (**b**) DNN.

The accuracy improved significantly when this fusion technique was adopted (See Table 11. The confusion matrices (Figure 12) along with the precisions and recalls were also generated for both algorithms in addition to the ROC (Figure 13) curve to obtain more insights into the result of combining both the spectrogram and GFCC.

## 5. Discussion

The first experiment shows that the GFCC features outperform the HR features in identifying the pathologic/healthy case. Moreover, after applying the combination/concatenation, this does not have much effect on the classification rate.

Trying to improve the model classification rate by applying hypertuning, it was possible to increase the accuracy of the optimal one for the SVM with the combined features to 94.79% and the SVM results were comparable with the results from the benchmark paper for the SVM of 95.92%, taking into account that they are classifying two classes while in this study, multiclassification is performed.

In addition, the precision, recall, and F1-score tables confirmed that the SVM on the combined data is the best, where it shows a high percentage above 90% for all categories. Where the precision, for example, for the RDS case, represents the proportion of infants identified as infected with RDS and was correct, for the recall it is the proportion of actual RDS that was identified correctly.

Moreover, reviewing the confusion matrix for the combined features of both the SVM and RF, we noticed that the SVM was more accurate in detecting the healthy cases rather than the pathological ones, while the RF was most accurate in detecting RDS.

The final experiment was to study the feature importance using the random forest feature importance as the GFCC features have a higher importance than the HR ones but the HR still has a relatively high importance. Note that it is an option here to drop any feature as each extracted feature represents a separate characteristic within the audio signals. As part of the evaluation, the ROC has been created and as this is a multiclassification problem, the ROC curve will have a different curve for each class. The same conclusion can be drawn for both the SVM and RF ROC, that the area under the curve is approximately the same for both the combined features and the GFCC while it is much less for the HR features which confirms the previous point as well. As can be seen from the results, combining the HR with the GFCC features does not have much effect on the classification rate.

This sounds reasonable as the GFCC has 14 different features while the HR has only 4, so it is clear that the GFCCs should have more information to depend on in the classification process. Moreover, the harmonic ratio features describe the relative strengths of different harmonic frequencies in a sound, which may not be as effective in implementing audio classification problems because they do not take into account other important characteristics of the sound, such as its temporal structure or the presence of noise. Additionally, the harmonic ratios of a sound can be affected by many factors, such as the recording quality and the specific instrument or voice producing the sound, which can make it difficult to use these features to accurately classify the sound.

This leads us to experiment with further audio domain features, for example, the spectrogram, which was the next step in this project, by feeding the extracted spectrogram images into a CNN model to extract the features to be combined with the GFCC feature and fed into a DNN model. Therefore, to make a further improvement, an additional audio feature domain was investigated which was the image domain through the spectrogram feature. This was examined separately and combined through the learning process of the DNN or by simple concatenation. The highest accuracy was achieved by the combination of the GFCC, HR, and spectrogram through the learning process at 97.50%, which can be seen from the ROC curve as well. The simple concatenation with DNN does not achieve much improvement in the classification rate which reaches 93.97% compared to 93.00% for the spectrogram as a standalone input. The confusion matrices imply that the system was able to classify the three categories with approximately similar precision and recall without any bias toward any of the three categories.

The results for the three types of features when experimented with separately show that the spectrogram outperforms the GFCC and HR as it can capture the time–frequency information of the audio signals that may be useful for classification tasks. In contrast, the GFCC and HR are different feature representations of audio signals that capture the spectral properties of the signal only. Therefore, the spectrogram representation provides a more discriminative feature representation compared to the GFCC or HR.

The findings show a promising result and demonstrate the importance of the feature fusion, specifically the GFCC, HR, and the spectrogram, through the learning process rather than the simple concatenation of the input features. The explanation is that when the simple concatenation was used, the 4096 features were concatenated with 13 GFCC and 4 HR features which reduce the effect of the GFCC and HR features. In contrast, through the learning process, the GFCC features were combined with the most important spectrogram features of 256, which makes the effect of these features more significant. Fusing features through the learning process as opposed to simple concatenation at the input stage obtained better classification results. Although the DNN in both cases can adjust the importance of each feature and determine how to best combine the features, this might be due to the fact that the GFCC and HR features interact better with the extracted 256 spectrogram features within the learning process. However, in the case of simple concatenation, the model only has a static representation of the input features. By fusing the features through the learning process, as recently proposed in [64], the model can learn the nonlinear relationships between the features and use this information to improve its overall performance. This results in a more robust model that is better suited to the specific task at hand and can result in improved accuracy and precision in the output, similar to the effect of using the CNN and DNN.

Moreover, the SVM with concatenated features and the Softmax used through the DNN gave a close accuracy which emphasized the DNN’s ability to generate a sparse code that can improve the split between the different categories. Therefore, the 4096 features concatenated with the GFCC/HR features by a simple concatenation and fed into the SVM model. The expectation is that both algorithms will result in very close results because the features have already been extracted and processed by a neural network, which can potentially capture complex nonlinear relationships in the data. Therefore, even though the SVM is a sophisticated model while SoftMax is a more statistical model, both will be able to achieve close classification rates.

The comparative Table 12 presented here evaluates the proposed model in the published paper [8] with the proposed model in this study. The table depicts a comprehensive comparison of several aspects of the two models, including the number of classes, audio features, feature fusion techniques, machine learning algorithms, and best accuracy details.

The proposed model in this study exhibits a more comprehensive diagnosis system as compared to the model proposed in the published paper [8]. Specifically, the model in this study includes an additional normal class in addition to RDS and sepsis, which provides a more balanced representation of the various conditions that can affect infants’ crying signals. Moreover, the proposed model in this study incorporates a more extensive set of audio features that can capture more nuanced aspects of the signals. While both models use GFCC and HR, the proposed model in this study also incorporates spectrogram features, which can provide additional information about the frequency content and temporal dynamics of the signals. Investigating spectrogram features is important because they can capture variations in the signal that are not well represented by other features, such as changes in pitch and modulation patterns.

Additionally, the proposed model employs both simple concatenation and fusion through the learning process as feature fusion techniques. This contrasts with the published paper which solely uses simple concatenation. Thus, it offers a potentially more effective approach to feature fusion, which can further enhance the accuracy of the diagnostic system. Investigating fusion through the learning process is important because it allows the model to learn how to combine the different features in a way that maximizes their diagnostic value, rather than relying on a fixed concatenation scheme.

In the modeling part, the proposed model has adopted a more diverse range of machine learning algorithms, including support vector machine (SVM), random forest (RF), and deep learning (DL). This compares to the model proposed in the published paper [8], which uses only a multilayer perceptron (MLP). Investigating deep learning is important because it allows the model to automatically learn hierarchical representations of the input data, which can capture complex patterns and interactions between the features. This can be particularly useful in a diagnostic system where the features may interact in nonlinear and complex ways. This diverse range of machine learning algorithms offers a more sophisticated and comprehensive approach to classification, which can lead to more accurate diagnoses.

The proposed model in this study achieves a higher accuracy of 97.50% as compared to the proposed model in the published paper [8], which achieves an accuracy of 95.92%. This indicates the superiority of the proposed model in this study in diagnosing pathologies in infants’ crying signals even after introducing a new class. Overall, the comparison table highlights the advancements and improvements made in this study as compared to the published paper [8]. The proposed model in this study offers a more comprehensive diagnosis system, employs a more extensive set of audio features, offers more effective feature fusion techniques, employs a diverse range of machine learning algorithms, and achieves a higher accuracy. The proposed model could significantly contribute to the early diagnosis and treatment of pathologies in infants’ crying signals, which can have significant implications for healthcare.

## 6. Conclusions

In this study, a medical diagnostic system for infants’ crying signals using audio domain feature fusion and DL algorithms is proposed for the early detection and discrimination of two pathologies associated with high mortality rates in newborns, neonatal respiratory distress syndrome (RDS) and sepsis. This study is performed upon the work of [8] by improving their dataset by adding a new class, which is the healthy one, and their methodology by considering additional features, which are the spectrogram features. Conventional DL and machine learning techniques were combined to create a system that performs comparably to more complex methods. The fusion of three different feature sets, specifically through the learning process, improved the overall performance of the system, resulting in an improvement in the accuracy and classification rates, where the highest accuracy of 97.50% was achieved by combining the spectrogram, HR, and GFCC features through the learning process which then fed into the DNN. As for future work, this study highlights the potential of using DL and audio domain feature fusion in the development of advanced diagnostic systems that can improve the accuracy and speed of medical diagnosis. This will include expanding the dataset to include a wider range of infant pathologies and developing a multimodal for the diagnosis of various infant conditions. Moreover, there will be an investigation of the preprocessing step, specifically the segmentation process, where we can study the effect of the segment length on the model performance. In addition, in future, we would like to address a limitation related to the data-splitting strategy used in this study. The data splitting was performed on the segmented data rather than at the individual infant level, primarily due to data collection challenges and the need for an adequate sample size. It is important to note that this approach may lead to an overestimation of the model’s performance on the testing data. Since samples from the same infants can be present in both the training and testing datasets, shared characteristics and patterns within an individual’s data may bias the evaluation of the model’s generalizability. Therefore, in future research, we will explore alternative data-splitting strategies. Splitting the data at the individual infant level could provide a more accurate evaluation of the model’s performance. In addition to an investigation of the integration of demographic features and additional audio features from other domains to further improve the performance of the system using different fusion techniques, we will also investigate various CNN models for feature extraction, for instance, ResNet.

## Figures and Tables

**Table 1:** Dataset Description for the Selected pathological cases.

Demographic Factors	Details
Gender	Female and Male
Babies‘ Ages	1 to 53 days old
Weight	0.98 to 5.2 kg
Origin	Canada, Haiti, Portugal, Syria, Lebanon, Algeria, Palestine, Bangladesh, and Turkey.
Race	Caucasian, Arabic, Asian, Latino, African, Native Hawaiian, Quebec.

**Table 2:** Dataset Description for the pathological cases.

Samples Category	Sepsis	RDS	Healthy
Number of samples after the preprocessing	2554	4369	9000
Number of samples selected randomly	(1132 each, in total 3396)		

**Table 3:** The evaluation measures and their formula.

Evaluation Measure	Formula
Accuracy	(TP + TN)/(TP + TN + FP + FN)
Precision	TP/(TP + FP)
Recall	TP/(TP + FN)
F1-score	2 × (precision × recall)/(precision + recall)

**Table 4:** ML algorithm accuracies without/with hypertuning.

ML Algorithm	GFCC		HR		HR and GFCC	
	Without Tuning	With Tuning	Without Tuning	With Tuning	Without Tuning	With Tuning
SVM	89.26%	94.47%	59.70%	63.24%	90.14%	**94.79%**
RF	89.85%	90.00%	60.73%	61.32%	90.14%	91.18%

**Table 5:** Support Vector Machine optimal hyperparameter combination after hyper-tuning using grid search.

Hyperparameter Name	Hyperparameter Values	Hyperparameter Optimal Value
C	[0.5, 1, 2, 4, 5]	2
gamma	[0.1, 0.25, 0.26, 0.3, 0.5]	0.5

**Table 6:** Random Forest optimal hyperparameter combination after hyper-tuning using grid search.

Hyperparameter Name	Hyperparameter Values	Hyperparameter Optimal Value
n_estimators	[50, 60, 80, 90]	90
max_depth	[70, 80, 90, 100, 120]	100
min_samples_split	[2, 5]	2
min_samples_leaf	[1, 2]	1

**Table 7:** Precision and Recall.

	Class	RF Combined	SVM Combined
Precision	Healthy	93.00%	95.00%
	RDS	90.00%	93.00%
	Sepsis	91.00%	94.00%
Recall	Healthy	92.00%	97.00%
	RDS	93.00%	92.00%
	Sepsis	89.00%	92.00%
F1-score	Healthy	92.46%	96.48%
	RDS	91.44%	92.68%
	Sepsis	90.46%	93.97%

**Table 8:** SVM best hyperparameter combination after hypertuning using grid search.

Hyperparameter Name	Hyperparameter Values	Hyperparameter Optimal Value
C	[2.0, 5.0, 10.0]	2.0
gamma	[‘linear’, ‘sigmoid’, ‘poly’, ‘RBF’]	linear

**Table 9:** DNN best hyperparameter combination after hypertuning using Keras.

Hyperparameter Name	Hyperparameter Values	Hyperparameter Optimal Value
Learning_rate	[0.0001, 0.001, 0.002]	0.001
weight_decay	[0.0, 0.0001, 0.001]	0.0

**Table 10:** Accuracy using spectrogram features and combined spectrogram and GFCC features.

ML Algorithm	Spectrogram		Spectrogram and GFCC		Spectrogram, GFCC, and HR	
	without Tuning	with Tuning	without Tuning	with Tuning	without Tuning	with Tuning
SVM	92.05%	92.94%	93.30%	93.97%	93.38%	93.38%
DNN (fusion through input concatenation)	93.00%	94.26%	93.97%	94.80%	93.82%	94.93%
DNN (fusion through learning)	93.00%	94.26%	95.44%	96.47%	96.17%	***97.50%***

**Table 11:** Precision and Recall.

	Class	DNN with Spectrogram HR and GFCC
Precision	Healthy	98.00%
	RDS	97.00%
	Sepsis	97.00%
Recall	Healthy	99.00%
	RDS	97.00%
	Sepsis	96.00%
F1-score	Healthy	98.49%
	RDS	97.00%
	Sepsis	96.48%

**Table 12:** Comparison between the model in [8] and the proposed model in this study.

Comparison	Model [8]		Proposed Model in This Study		
Classes	RDS, sepsis		RDS, sepsis, normal		
Audio features	GFCC, HR		GFCC, HR, spectrogram		
Feature fusion Techniques	Simple concatenation		Simple concatenation and fusion through the learning process		
ML algorithm	MLP	SVM	RF	SVM	DL
Best accuracy details	GFCC, HR using simple concatenation	GFCC, HR using simple concatenation	GFCC, HR using simple concatenation	GFCC, HR using simple concatenation	GFCC, HR, spectrogram using fusion through the learning process
Accuracy	95.92%	92.94%	94%	91.18%	97.50%

## Data Availability

Not applicable.
